# Associations of Brain Reactivity to Food Cues with Weight Loss, Protein Intake and Dietary Restraint during the PREVIEW Intervention

**DOI:** 10.3390/nu10111771

**Published:** 2018-11-15

**Authors:** Mathijs Drummen, Elke Dorenbos, Anita C. E. Vreugdenhil, Gareth Stratton, Anne Raben, Margriet S. Westerterp-Plantenga, Tanja C. Adam

**Affiliations:** 1 Department of Nutrition and Movement Sciences, Maastricht University Medical Centre, 6200 MD Maastricht, The Netherlands; t.adam@maastrichtuniversity.nl; 2 NUTRIM School of Nutrition and Translational Research in Metabolism, Maastricht University, 6200 MD Maastricht, The Netherlands; elke.dorenbos@mumc.nl (E.D.); a.vreugdenhil@mumc.nl (A.C.E.V.); m.westerterp@maastrichtuniversity.nl (M.S.W.-P.); 3 Centre for Overweight Adolescent and Children’s Health Care (COACH), Department of Paediatrics, Maastricht University Medical Centre, 6200 MD Maastricht, The Netherlands; 4 Research Centre in Applied Sports, Technology Exercise and Medicine, College of Engineering, Swansea University, Swansea, SA1 8EN Wales, UK; g.stratton@swansea.ac.uk; 5 Department of Nutrition, Exercise and Sports, University of Copenhagen, DK-1017 Copenhagen, Denmark; ara@nexs.ku.dk

**Keywords:** fMRI, food cues, food reward, obesity, insulin resistance, protein intake, weight loss

## Abstract

The objective was to assess the effects of a weight loss and subsequent weight maintenance period comprising two diets differing in protein intake, on brain reward reactivity to visual food cues. Brain reward reactivity was assessed with functional magnetic resonance imaging in 27 overweight/obese individuals with impaired fasting glucose and/or impaired glucose tolerance (HOMA-IR: 3.7 ± 1.7; BMI: 31.8 ± 3.2 kg/m^2^; fasting glucose: 6.4 ± 0.6 mmol/L) before and after an 8-week low energy diet followed by a 2-year weight maintenance period, with either high protein (HP) or medium protein (MP) dietary guidelines. Brain reactivity and possible relationships with protein intake, anthropometrics, insulin resistance and eating behaviour were assessed. Brain reactivity, BMI, HOMA-IR and protein intake did not change differently between the groups during the intervention. In the whole group, protein intake during weight maintenance was negatively related to changes in high calorie images>low calorie images (H > L) brain activation in the superior/middle frontal gyrus and the inferior temporal gyrus (*p* < 0.005, corrected for multiple comparisons). H > L brain activation was positively associated with changes in body weight and body-fat percentage and inversely associated with changes in dietary restraint in multiple reward, gustatory and processing regions (*p* < 0.005, corrected for multiple comparisons). In conclusion, changes in food reward-related brain activation were inversely associated with protein intake and dietary restraint during weight maintenance after weight loss and positively associated with changes in body weight and body-fat percentage.

## 1. Introduction

Overweight and obesity are established risk factors for the development of type 2 diabetes (T2D) [1] and insulin resistance [2]. Both, obesity and T2D have been associated with neuronal alterations in areas associated with reward processing [3,4,5]. Comparing obese and normal-weight individuals, functional neuroimaging studies have reported increased anticipatory brain activation in response to visual food cues in gustatory and reward regions (including the striatum, insula, amygdala, orbitofrontal cortex and hippocampus) [6]. In response to food consumption however, obese individuals showed reduced activation of brain reward circuits compared to normal-weight individuals, suggesting decreased inhibitory control [4].

Along similar lines, patients with T2D showed increased brain activation in response to viewing food images in the insula, caudate and orbitofrontal cortex [5] and a lack of inhibition of hypothalamic neuronal activity after glucose ingestion [7] compared to healthy controls. Central insulin resistance was suggested to possibly play a role in the altered brain activation in both, obesity and T2D. In a study measuring cerebral blood flow, a blunted response in the prefrontal cortex was observed after intranasal insulin administration in obese men, which was inversely related to peripheral insulin sensitivity, independently of BMI [8]. Further evidence comes from studies finding an inverse relationship between markers of peripheral insulin sensitivity and brain activation in response to food cues in brain reward regions [9,10]. In line with these findings, we previously reported positive associations between insulin resistance and reactivity to food cues in the insula, anterior cingulate gyrus and nucleus accumbens in overweight and obese individuals with impaired fasting glucose and/or impaired glucose tolerance [11]. Given that insulin binds to receptors expressed on neurons in areas of reward processing [12], central insulin resistance may contribute to impaired reward signalling and a reduction in insulin resistance may help to normalize the reward response.

One of the most potent strategies to reduce insulin resistance is body weight loss. It has been shown that food-cue reactivity of reward regions was also suppressed along with extended caloric restriction [13]. Patients with T2D who underwent bariatric surgery showed decreased reward activation along with a normalization in glycaemic control, compared to T2D patients without surgery and impaired glycaemic control [14]. Besides surgery, lifestyle intervention is a tool to reduce weight and increase insulin sensitivity. The intervention requires profound changes in eating behaviour but it remains unclear how changes in aspects of eating behaviour, such as cognitive restraint, relate to changes in neuronal activation.

Considering the well-established effect of protein on satiety, diet induced thermogenesis and the preservation of fat free mass during weight loss, it seems to be the intuitive macronutrient in the nutritional component of a lifestyle intervention. While largely unknown, the beneficial effects of protein for weight loss and weight maintenance may in part be mediated through an effect on reward signalling [15]. In the rat, brain signalling was reduced in the amygdala after infusion of an intragastric protein load [16]. Human short-term studies also reported a reduction in brain activation to food cues and subsequent reductions in energy [17,18] or changes in food preference [19] after protein ingestion.

The aim of the current study was to investigate the effects of weight loss and subsequent weight maintenance with higher protein intake versus moderate protein intake on brain reward activity in response to visual food cues in participants of the PREVIEW study (Prevention of diabetes through lifestyle intervention in population studies in Europe and around the World). The PREVIEW study aims to find the most effective lifestyle for the prevention of T2D in overweight and obese participants with increased risk for type 2 diabetes (www.previewstudy.com). We hypothesized that participants with higher protein intake had reduced brain reactivity to food cues after 2 years compared to participants with moderate protein intake.

## 2. Materials and Methods

### 2.1. Participants

Forty participants were recruited from the PREVIEW-cohort at Maastricht University and a total of twenty-seven participants completed both fMRI measurements. Inclusion criteria were: age 25–70 years, BMI ≥ 25 kg/m^2^, fasting plasma glucose of 5.6–6.9 mmol/L and/or plasma glucose concentration of 7.8–11.0 mmol/L at 2 h after an oral glucose tolerance test (OGTT) and willingness to undergo MRI procedures. Exclusion criteria included T2D, left-handedness, claustrophobia and history of neurological disorders in addition to the general PREVIEW exclusion criteria (ClinicalTrials.gov NCT01777893). The study was approved by the Medical Research Ethics Committee of Maastricht University Medical Centre and in accordance with guidelines of the Declaration of Helsinki. All participants provided written informed consent for participation.

Participants started with 8 weeks on a low energy diet (LED), followed by a weight maintenance period with instructions to follow dietary guidelines in two groups, a moderate protein (MP) and high protein (HP) group. Only participants that reached at least 8% weight loss with the LED were allowed to continue in the weight maintenance period. Five participants dropped-out during the LED period, seven participants were lost to follow-up during the weight maintenance period. One participant lacked valid functional imaging data and had to be excluded from analysis. After this, twelve participants completed the study in the MP group and fifteen in the HP group (Supplemental Figure S1).

### 2.2. Measurements

Measurements included functional magnetic resonance imaging (fMRI), body composition, blood sampling before and during an oral glucose tolerance test (OGTT) and behavioural questionnaires. Prior to the investigation days, participants collected 24-h to determine nitrogen excretion as a marker for protein intake. Accelerometers were worn for 7 days to calculate physical activity. At baseline and after 2 years, measurements were performed after an overnight >10 h fasting period.

### 2.3. Study Design

The LED provided 3.4 MJ (35–40 E% protein, 45–50 E% carbohydrate, 15–20 E% fat) per day with four sachets of the Cambridge Weight Plan^®^, 3 of which were dissolved in 250 mL low fat milk and one in 250 mL water. Additionally, energy-free drinks and <400 gram per day of non-starchy, low-CHO vegetables were allowed. Participants were randomized into two dietary intervention groups at the start of the study but received no information regarding their intervention group until after the LED phase. Dietary intervention groups comprised a moderate protein (MP) group with 15/55/30% of energy from protein/carbohydrate/fat and a moderate dietary glycaemic index (GI) (≥56) and a high protein (HP) group with 25/45/30% of energy from protein/carbohydrate/fat, with a low dietary GI (≤50). Both diets were consumed ad libitum with respect to energy but with the instruction to maintain the achieved body weight. Additional weight loss was allowed. Participants were given examples of daily eating plans according to the macronutrient and GI requirements of the two intervention groups. More detailed information on the dietary guidelines and of the intervention groups has been reported before [20].

### 2.4. Brain Imaging Paradigm and Acquisition

Participants underwent functional magnetic resonance imaging to obtain blood-oxygen level-dependent (BOLD) data. Scanning was performed on a 3 Tesla scanner (Magneto, Siemens, Erlangen, Germany) using a 64-channel head coil. Nine randomized blocks of ten images were presented during scanning and blocks contained images of high-calorie foods, low-calorie foods or non-food objects. Images were chosen from the International Affective Picture System [21] and various web sites, which has been described previously [11]. High-calorie images included items such as fries, mac-and-cheese, hamburgers, donuts and so forth. Low-calorie images included items such as fruit salad, cucumbers, carrots, broccoli and so forth. Food images were randomly shown from sixty different images in order to avoid preference and learning effects. Images were shown for 2 s each. Between the blocks, a fixation cross was shown for 10 s. Participants received the instruction to focus on how much they liked the images. Functional runs were collected using a T2*-weighted protocol (TR = 2.0 s, TE = 30.0 ms, FOV = 216 × 216, matrix size = 72 × 72, voxel size, 3 × 3 × 3 mm^3^). Each volume consisted of 34 slices, acquired in interleaved ascending order. In each session, anatomical images were acquired with a high-resolution T1 weighted scan.

### 2.5. Image Processing

Analysis of the fMRI data was performed with Brain Voyager 20.6 (Brain Innovation B.V, Maastricht, The Netherlands). Pre-processing of the functional images included slice scan time correction with cubic spline interpolation, 3D motion correction and temporal high-pas filtering with 4 cycles. Anatomical data was corrected for intensity inhomogeneities. The anatomical scan of each participant was transformed into Montreal Neurological Institute (MNI) space. After pre-processing, both functional and anatomical scans were aligned based on starting position and boundary-based registration. The volume-time-courses were spatially smoothed by applying a Gaussian filter (FWHM = 6) to increase signal-to-noise ratio. For each participant, food reward-related brain activation predictors for the three stimuli blocks (high-calorie food images, low-calorie food images and neutral images) were applied in Generalized Linear Model (GLM) analyses and the hemodynamic-response was taken into account by adding a Two Gamma Hemodynamic Response Function and movement parameters were modelled as confounding predictors. To investigate the two scan sessions dummy coding was applied with the BVA-Predictor Tool software (BVA-Predictor Tool, J.M. Born, Maastricht, The Netherlands). Region of interest (ROI) analysis was used to specifically investigate the activation in the right and left nucleus acumens, the right anterior cingulate gyrus and right insula. Regions were selected based upon previous research, which identified food reward related brain activation in these regions to be correlated with insulin resistance [11]. See Supplemental Figure S2 for a visualization of the ROIs.

### 2.6. Body Weight and Composition

Body weight was measured using a calibrated scale (Life Measurement Corporation, Inc., Concord, CA, USA) and body composition was determined using the BodPod System (Life Measurement Corporation, Inc., Concord, CA, USA) [22]. Height was measured using a wall-mounted stadiometer to the nearest 0.1 cm (Seca, model 222, Seca, Hamburg, Germany).

### 2.7. Blood Samples and Urinary Nitrogen Excretion

Blood samples were taken to determine fasting glucose and insulin concentrations. The homeostatic model assessment for insulin resistance (HOMA-IR) was used to assess insulin resistance and was calculated as follows: fasting glucose × fasting insulin / 22.5 [23]. Blood samples were analysed at the National Institution for Health and Welfare in Helsinki, Finland. Plasma glucose was measured by enzymatic hexokinase method and insulin was measured using chemiluminescent microparticle immunoassay. Nitrogen excretion was measured from 24-h urine collections, as a marker of protein intake. Urine was collected in 2 L urine bottles that contained 10 mL 4M hydrochloric acid to prevent nitrogen loss. Nitrogen concentration was measured with a nitrogen analyser (CHO-O-Rapid; Hereaus, Hanau, Germany) and multiplied with the total volume to determine nitrogen excretion per day. Nitrogen excretion per day was multiplied by 6.25 and divided by the body weight to determine daily oxidation per kilogram body weight. 24-h urine was collected at baseline, after 6 months, after 1 year and after 2 years. Averages of the 6 month, 1 year and 2 year samples were used as a representation of protein intake during the weight maintenance period.

### 2.8. Physical Activity

Physical activity was assessed using the ActiSleep+ accelerometer (Actigraph LLC, Pensacola, FL, USA). Participant wore the accelerometer for seven consecutive days on the right side of an elastic belt around the waist. Data was collected at 100 Hertz and aggregated to 60 s epochs. After removal of sleep episodes, average counts per minute (CPM) were calculated.

### 2.9. Eating Behaviour

Eating Behaviour was assessed using the Three Factor Eating Questionnaire (TFEQ), which consists of 51 questions [24]. The three factors it measures are cognitive restraint (TFEQ factor 1), emotional eating and disinhibition (TFEQ factor 2) and feelings of hunger (TFEQ factor 3).

### 2.10. Statistical Analysis

Data are expressed as mean ± SD SPSS 23 (IBM Corp., Armonk, NY, USA). Variables were tested for normality and log-transformed if applicable. Factorial ANOVA was used to assess differences between groups at baseline. Two-way repeated measures ANOVA was used to test differences between groups over time. Repeated-measures ANOVA was used to test differences over time for the whole group. Two-tailed *p* values <0.05 were considered statistically significant. For the fMRI data, contrasts of interest from single-subject analysis were tested in GLM analyses and then submitted to second-level whole brain random effect analysis to compare groups at baseline or changes between groups. Contrasts included food (high calorie images + low calorie images) versus non-food (neutral images) (F > nF) and high calorie images versus low calorie images (H > L). Whole brain correlation analyses was used to test the correlation between changes in F > nF or H > L reactivity and changes in anthropometric and behavioural parameters over the intervention period in the whole group of participants. Monte Carlo simulations were performed in BrainVoyager to identify cluster-extent thresholds with a cluster alpha of 0.005, to correct for multiple comparisons. Simulations were performed with 1000 iterations and an independent voxel threshold of *p* < 0.001. Regarding the ROI analysis, average parameter estimates within each ROI were extracted and exported to SPSS to compare differences between groups. Pearson’s correlation analysis was used to determine the relation between brain reactivity within significant clusters or ROIs and anthropometric and behavioural parameters. Partial correlation analysis was used to determine the relationship between anthropometric and behavioural parameters and extracted brain activation in the significant clusters or ROIs adjusted for changes in BMI. There were no differences in changes in brain activation between men and women. A *p* < 0.005 was considered significant to correct for multiple comparisons. Analyses were performed using BrainVoyager 20.6 (Brain Innovation B.V., Maastricht, The Netherlands) and the Statistical Package for the Social Sciences (SPSS, IBM Corp., IBM SPSS Statistics, V23, Armonk, NY, USA).

## 3. Results

Characteristics of the participants in the two groups are shown in Table 1. Regarding anthropometrics, insulin resistance, protein intake and physical activity (CPM), no differences were observed at baseline, nor in changes during the intervention between the groups. All anthropometric parameters decreased significantly (see Table 1) in the whole group during the intervention period (*p* < 0.001). Fasting insulin, glucose, c-peptide, HbA1c concentrations and HOMA-IR significantly decreased (*p* < 0.05). Physical activity (CPM) did not significantly change during the intervention. During the intervention TFEQ restraint increased for the whole group. TFEQ hunger significantly decreased in the whole group of participants (Table 1).

### 3.1. No Differences in Brain Activity Between MP and HP Groups

There were no differences in brain activation in response to viewing food versus non-food images (F > nF) or to high-calorie versus low-calorie images (H > L) between the groups at baseline. Differences in brain activation over the intervention period were compared between the two groups using whole brain analysis. There were no differences in changes for the contrasts food versus non-food images or high-calorie versus low-calorie images between the groups. Furthermore, extracted brain activation in the *a priori* defined ROIs did not differently change between the groups for the contrasts food versus non-food images or high-calorie versus low-calorie images.

During the intervention, daily nitrogen excretion or daily protein intake were not different between the groups. Therefore, both groups were taken together for further analyses. In the whole group of participants, daily nitrogen excretion (12.1 ± 4.1 g to 13.8 ± 4.4 g) and daily protein intake 0.82 ± 0.23 g/kg to 1.04 ± 0.25 g/kg) were increased at 2 years compared to baseline (*p* < 0.05). During the weight maintenance period, average daily nitrogen excretion was 14.2 ± 3.9 g and average protein intake was 1.06 ± 0.24 g/kg.

### 3.2. Whole Brain Analysis-Relations Between Brain Activation and BMI, Body-Fat Percentage, HOMA-IR, Protein Intake, Activity or Eating Behaviour

For the whole group, brain activation for the contrast food versus non-food images or high-calorie versus low-calorie images was not significantly different at 2 years compared to baseline, assessed with whole brain analyses. To determine whether changes in brain reactivity to food cues were associated with anthropometrics, insulin resistance, protein intake, activity or eating behaviour whole brain analyses with changes in BMI, body-fat percentage, HOMA-IR, protein intake (g/kg), physical activity (CPM) or TFEQ scores added as covariates were performed for the whole group (Table 2) (Supplemental Figures S2–S6).

#### 3.2.1. Food > Non-Food Brain Response

Changes in body-fat percentage were negatively associated with changes in food versus non-food images activation in the gyrus rectus and positively associated with changes in food versus non-food images activation in the left thalamus and left middle frontal gyrus. There were no significant clusters for changes in food versus non-food images activation and changes in other variables.

#### 3.2.2. High Calorie > Low Calorie Brain Response

**Figure 1:**
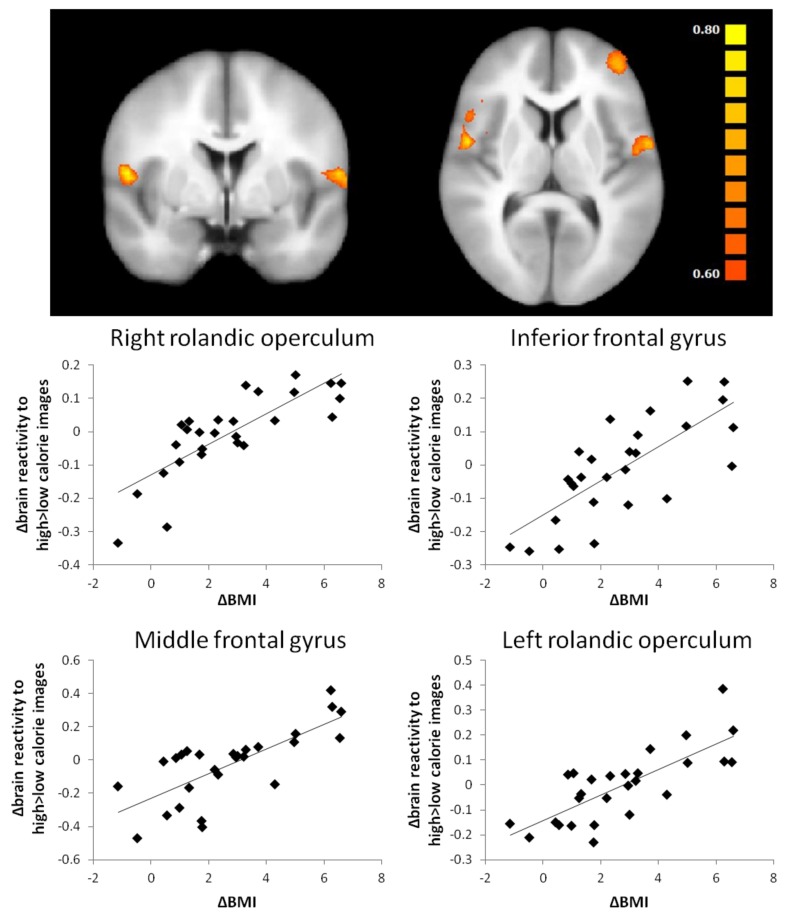
Whole brain contrast map of brain regions with significant associations between changes in high > low calorie images brain activation and changes in BMI. Positive associations are shown in orange (*p* < 0.005, corrected for multiple comparisons). Scatter plots of changes in BMI and changes in extracted high > low calorie images BOLD response are shown below.

**Figure 2:**
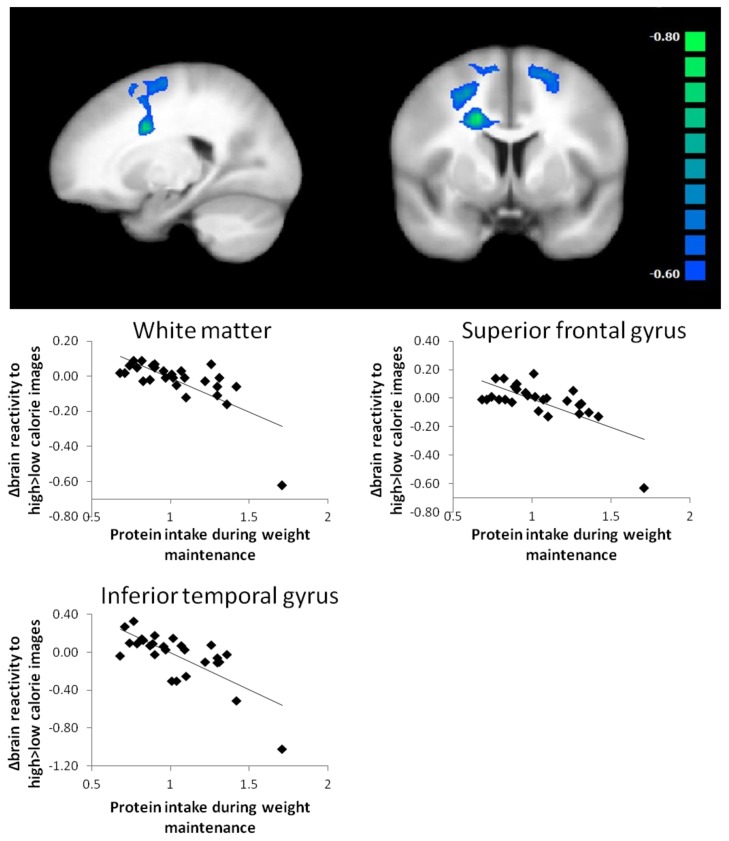
Whole brain contrast map of brain regions with significant inverse associations between changes in high > low calorie images brain activation and daily protein intake (g/kg) during weight maintenance. Inverse associations are shown in blue (*p* < 0.005, corrected for multiple comparisons). Scatter plots of protein intake and changes in extracted high > low calorie images BOLD response are shown below.

Changes in BMI were positively related to changes in high-calorie versus low-calorie images brain activation in regions associated with reward and control (Figure 1) (rolandic operculum, inferior frontal gyrus, middle frontal gyrus). Changes in body-fat percentage were positively related to changes in H > L brain activation in regions associated with visual processing, memory, reward and memory (middle temporal gyrus, angular gyrus, putamen, superior frontal gyrus, superior occipital gyrus, insula). Daily protein intake (g/kg) during weight maintenance was negatively related to high-calorie versus low-calorie images brain activation in the superior frontal gyrus and left inferior temporal gyrus (Figure 2). For high-calorie versus low-calorie images activation, changes in TFEQ dietary restraint (factor 1) were negatively associated with activation in the right superior temporal gyrus, the right precentral gyrus and the left and right superior occipital gyrus. There were no significant clusters for changes high-calorie versus low-calorie images brain activation and changes in HOMA-IR, CPM, TFEQ disinhibition or TFEQ hunger.

### 3.3. ROI Analysis-Relations between Brain Activation and BMI, Fat Mass, HOMA-IR, Protein Intake, Activity or Eating Behaviour

To specifically investigate the association in the *a priori* defined ROIs, average brain activation was extracted and associated with changes in BMI, fat mass, HOMA-IR, protein intake (g/kg), physical activity (CPM) or TFEQ scores.

#### 3.3.1. Food > Non-Food Brain Response

Adjusted for changes in BMI, changes in HOMA-IR were positively related to changes in food versus non-food images brain activation in the left nucleus accumbens (*r* = 0.60; *p* = 0.001). Changes in food versus non-food images brain activation in the ROIs were not associated with changes in BMI, body-fat percentage, CPM, eating behaviour or protein intake during weight maintenance.

#### 3.3.2. High Calorie > Low-Calorie Brain Response

For the high-calorie versus low-calorie images contrast, there was a positive association between changes in BMI and brain activation in the right insula (*r* = 0.62; *p* = 0.001) and right anterior cingulate (*r* = 0.58; *p* = 0.002). Adjusted for changes in BMI, there were positive associations between changes in body-fat percentage and brain activation in the left insula (*r* = 0.71; *p* < 0.001), the right (*r* = 0.54; *p* = 0.005) and left nucleus accumbens (*r* = 0.58; *p* = 0.002). Changes in CPM, TFEQ scores and protein intake during weight maintenance were not associated with changes in high-calorie versus low-calorie images brain activation.

## 4. Discussion

The aim of this study was to investigate the effects of weight loss and subsequent weight maintenance with relatively increased protein intake on brain reward activity in response to visual food in two groups of participants with overweight/obesity and IFG/IGT. There were no differences between the two protein groups in brain reactivity to food cues. However, we did not find differences in protein intake between the groups determined with urinary nitrogen excretion, implying the intervention was not successful in inducing the deliberate difference in protein intake between the groups.

Therefore, we applied post-hoc analyses on the whole group of participants to assess effects of protein intake on brain reactivity to food cues. Protein intake during the weight maintenance period in the whole group was negatively related to changes in brain activation contrasting high-calorie with low-calorie images in regions associated with frontal control (the superior frontal gyrus) and visual processing (the left inferior temporal gyrus). This is in line with evidence coming from short-term studies [18,19]. A high-protein breakfast was shown to further reduce hippocampal and parahippocampal reactivity to food cues compared to a normal-protein breakfast, measured in 20 overweight/obese girls in a randomized crossover study [18]. Moreover, in 23 healthy women a 12-day low-protein diet compared to a high-protein diet led to an increased BOLD response in the orbitofrontal cortex and striatum in response to food cues [19]. Based on our data, it is not possible to determine the causality of the relation between protein intake and brain reactivity to food cues but multiple explanations have been postulated. Protein intake may influence brain signalling via changes in the neurotransmitter precursor availability. The amino acid tryptophan is the precursor for serotonin and tyrosine and phenylalanine are precursors for dopamine [25]. Ingestion of protein increases the availability of these precursors, potentially resulting in higher neurotransmitter synthesis [26,27]. Furthermore, protein was shown to modulate food reward via activation of the vagus nerve, which projects to the nucleus tractus solitaries in the brain stem to relay satiety signals to the brain. Glutamate has shown to activate branches of the vagus nerve [28] and ingestion of glutamate led to activation of the amygdala, hippocampus and lateral hypothalamus in rats [29] while activation was fully suppressed after vagotomy. Indirectly, amino acids could also influence brain reward signalling through an increased release of anorectic hormones from the gut [15]. It needs to be mentioned however, that in our particular study, the effects expected to be due to increased protein intake, could also be related to decreased carbohydrate or fat intake during the intervention [30]. Increased protein intake during the dietary intervention may be part of a general change of a healthier lifestyle and other factors advocated in the PREVIEW intervention guidelines aiming at a generally healthier lifestyle could contribute to the results.

As expected, participants significantly reduced their body weight and fat mass during the intervention period. Changes in BMI were positively related to changes in brain reactivity to high-calorie compared to low-calorie images in the rolandic operculum, inferior frontal gyrus and middle frontal gyrus. Especially the inferior frontal gyrus and middle frontal gyrus have been implicated in dietary self-control [31,32]. The association with reactivity to high-calorie images versus low-calorie images is in line with studies reporting that activation in these regions modulates value-encoding regions when participants choose healthy over unhealthy foods [33]. A positive relationship between brain response to food versus non-food cues in the anterior cingulate and BMI was also found in a study by Martens et al. [4]. Furthermore, weight loss using a 6-month lifestyle intervention in overweight/obese individuals has previously shown to reduce brain activation to high-calorie images and cause a shift in relative activation favouring low-calorie versus high-calorie food cues in the putamen [34]. Weight loss after bariatric surgery was shown to support reduced neuronal responses to food cues in multiple food reward regions [14,35]. In our study, changes in body fat percentage were positively related to changes in brain activation contrasting high-calorie with low-calorie images in various gustatory and frontal control regions, including the putamen and insula. This may be interesting in terms of linking eating behaviour and body-fat mass [36]. Higher cue reactivity in the putamen and insula has been associated with food cravings and may be a mechanism to match higher energy needs with increased adiposity [10,37]. Our findings are in line with a study that reported a positive association between brain activation to high-calorie food cues and abdominal fat in girls, independent of BMI [38]. Suggested mechanisms through which fat mass may influence brain reward signalling are increased leptin concentrations or increased low-grade inflammation. Prior research reported a positive association between leptin concentrations and brain responses to high-calorie food versus non-food cues in the putamen/caudate, insula and amygdala in lean and obese individuals [39,40]. Leptin injections were able to reduce weight loss-induced effects on neural activity to visual food cues in brain areas associated with regulatory, emotional and cognitive control of food intake [41]. Adiposity-related inflammation has been suggested as another mechanism via which increased fat mass may moderate brain reward. Increased inflammation was shown to be related to reduced integrity and lower functional connectivity in food reward-related brain areas [42] but also to decreases in dopamine availability and release, leading to alterations in motivation and reward [43]. Changes in body-fat percentage were positively associated with changes in reactivity to high-calorie images versus low calorie-images in the putamen and insula.

Region-of-interest analysis revealed that in our participants, changes in peripheral insulin resistance were positively related to changes in food versus non-food brain activation in the left nucleus accumbens. These results follow up on findings of a previous study in which we reported brain activation to food cues to be positively related to HOMA-IR [11]. This observation is corroborating other studies reporting positive relations between markers of insulin resistance and brain reward signalling in different study populations [8,9,10]. In the present study we used HOMA-IR as a marker insulin resistance but our results are also supported by studies investigating the effects of intranasal insulin administration on brain reward signalling [44]. Intranasal insulin administration was shown to inhibit forward projections from the ventral tegmental area to the nucleus accumbens and this was related to a reduction in food value but these modulatory effects of insulin were not present in participants with increased insulin resistance [45]. We found a significant relation between changes in insulin resistance and changes in brain reward signalling over a two year period, further illustrating the importance of reducing insulin resistance.

With regard to eating behaviour, we found that changes in high versus low-calorie images were negatively associated with changes in dietary restraint in gustatory and visual processing regions. Dietary restraint increased during the intervention, which converges with studies reporting increased dietary restraint after caloric restriction [46,47]. This relation between dietary restrained and brain response to food images could be a possible mechanism via which dietary restraint leads to more healthy food habits [48,49].

Our study is limited by the lack of difference in protein intake between the two intervention groups. Due to this, we cannot exclude that the lack of differences in changes in brain reactivity between the intervention groups may be due to type II errors. Therefore, post-hoc analyses were performed on the whole group to assess the relation between protein intake and brain reactivity to food cues. We acknowledge the need of replicating our results in long-term studies with provided dietary protein in order to achieve the goals of protein intake levels as indicated.

## 5. Conclusions

In conclusion, we found an association between the relatively increased intake of protein and a reduction in brain response to high-calorie food images compared to low-calorie food images in brain reward related regions in the total group of participants. Changes of anthropometric parameters, including body weight and body fat percentage during the intervention period, were positively associated with changes in brain responses to high-calorie versus low-calorie images. The reduction in insulin resistance throughout the intervention period was associated with a general reduction in the food reward response, evidenced by the positive association of brain activation contrasting food versus non-food images. Changes in dietary restraint were inversely associated with changes in brain response to high-calorie food images compared to low-calorie food images in gustatory and visual processing regions.

## Figures and Tables

**Table 1:** Participant characteristics.

	MP (*n* = 12)	2 Years	HP (*n* = 15)	2 Years	Total (*n* = 27)	2 Years
	Baseline		Baseline		Baseline	
Age	54.7 (10.6)		52.7 (10.3)		53.6 (10.3)	
Height	1.74 (0.1)		1.69 (0.12)		1.71 (0.12)	
Weight (kg)	99.4 (17.4)	90.6 (16.0)	89.7 (15.4)	81.9 (15.6)	94.0 (16.7)	85.8 (16.1) ***
BMI (kg/m^2^)	32.7 (3.1)	29.9 (3.5)	31.2 (3.3)	28.5 (4.1)	31.8 (3.2)	29.1 (3.8) ***
FM	40.9 (8.8)	33.9 (12.0)	35.8 (8.7)	29.7 (10.9)	38.1 (9.0)	31.6 (11.4) ***
Body-fat %	41.4 (6.7)	37.2 (10.1)	40.2 (7.3)	36.2 (9.9)	40.7 (6.9)	36.6 (9.8) ***
Insulin	13.0 (6.4)	10.4 (5.5)	14.6 (6.5)	10.4 (3.7)	13.9 (6.4)	10.4 (4.5) **
Glucose	6.4 (0.8)	6.0 (0.7)	6.3 (0.5)	5.9 (0.4)	6.4 (0.6)	5.9 (0.5) **
HOMA-IR	3.7 (1.9)	2.8 (1.8)	4.2 (2.1)	2.7 (1.1)	4.0 (2.0)	2.8 (1.4) *
CPM	302.4 (116.1)	262.2 (97.4)	380.1 (109.3)	338.5 (116.8)	345.2 (116.3)	304.2 (112.6)
TFEQ f1	7.7 (4.4)	13.6 (2.8)	8.9 (4.8)	13.1 (3.5)	8.3 (4.5)	13.3 (3.1) ***
TFEQ f2	7.5 (3.3)	6.4 (2.3)	8.6 (3.7)	7.5 (4.0)	8.1 (3.5)	7.0 (3.4)
TFEQ f3	5.7 (3.0)	4.1 (3.1)	5.9 (3.6)	5.0 (3.6)	5.8 (3.3)	4.6 (3.3) *

Data are presented as mean ± standard deviation. Differences over time between MP and HP were assessed by means of factorial repeated-measures ANOVA; none were found. Changes over time within groups were determined using repeated-measures ANOVA, * *p* < 0.05, ** *p* < 0.01, *** *p* < 0.001. Abbreviations: MP: moderate protein group; HP: high protein group; HOMA-IR: homeostatic model assessment for insulin resistance; CPM: counts per minute, TFEQ: Three factor eating questionnaire, f1: factor 1 (cognitive restraint), f2: factor 2 (disinhibition and emotional eating), f3: factor 3 (feelings of hunger).

**Table 2:** Summary of whole brain fMRI results.

Contrast	Variable	AAL	k	*r*	*r* *	*p* *	x	y	z	Cluster Extent
ΔF > nF	ΔBody-fat%	gyrus rectus L	540	−0.66	−0.54 *	0.005 *	−1	55	−16	906
		thalamus L		0.64	0.62 *	0.001 *	−20	−22	13	1010
		no label (white matter L)		−0.63	−0.72 *	<0.001 *	−20	−13	38	579
		middle frontal gyrus L		0.63	0.65 *	<0.001 *	−24	23	41	1314
ΔH > L	ΔBMI	rolandic operculum R	621	0.66	-	-	55	0	9	688
		inferior frontal gyrus R		0.63	-	-	47	17	3	1807
		middle frontal gyrus L		0.66	-	-	−41	50	8	1610
		rolandic operculum L		0.65	-	-	−58	−2	8	796
	ΔBody-fat %	angular gyrus R	675	0.65	0.71 *	<0.001 *	61	−51	34	2881
		middle temporal gyrus R		0.62	0.72 *	<0.001 *	48	1	−25	893
		putamen R		0.65	0.68 *	<0.001 *	31	6	−1	11,852
		superior frontal gyrus L		0.65	0.65 *	<0.001 *	−15	59	16	8828
		superior occipital gyrus R		0.64	0.57 *	0.003 *	17	−86	21	1269
		insula L		0.65	0.71 *	<0.001 *	−40	14	2	8394
	WM protein intake (g/kg)	no label (white matter R)	405	−0.63	−0.71 *	<0.001 *	20	4	49	4040
		superior frontal gyrus L		−0.64	−0.67 *	<0.001 *	−16	9	59	2398
		inferior temporal gyrus L		−0.64	−0.72 *	<0.001 *	−65	−34	−18	1062
	ΔTFEQ f1	superior temporal gyrus R	569	−0.65	−0.69	<0.001 *	59	−44	15	1835
		precentral gyrus R		−0.67	−0.72	<0.001 *	51	6	38	1127
		superior occipital gyrus R		−0.64	−0.64	0.001 *	28	−79	23	601
		superior occipital gyrus L		−0.66	−0.80	<0.001 *	−21	−79	33	1014

Notes: Cluster extent threshold was determined by Monte Carlo simulation with a voxel threshold of *p* < 0.001 and cluster alpha <0.005. Centre-of-gravity coordinates of each cluster are given in MNI space and the automated anatomical labelling atlas was consulted to obtain corresponding anatomical labels. * corrected for ΔBMI. Abbreviations: k, cluster threshold in mm^3^; AAL, automated anatomical labelling; F > nF food images versus non-food images; H > L high calorie images versus low calorie images; R, right hemisphere; L, left hemisphere; TFEQ, three factor eating questionnaire, f1 = factor 1 (cognitive restraint).

## Supplementary Materials

The following are available online at http://www.mdpi.com/2072-6643/10/11/1771/s1, Figure S1: Participant flowchart, Figure S2: *A priori* selected regions of interest, Figure S3: Whole brain contrast map of brain regions with significant associations between changes in food > non-food brain activation and changes in body-fat percentage, Figure S4: Whole brain contrast map of brain regions with significant inverse associations between changes in food > non-food brain activation and changes in body-fat percentage, Figure S5: Whole brain contrast map of brain regions with significant associations between changes in high > low calorie images brain activation and changes in body-fat percentage, Figure S6: Whole brain contrast map of brain region with significant inverse associations between changes in high > low calorie images brain activation and changes in TFEQ dietary restraint (factor 1).

## References

[1] Vazquez G., Duval S., Jacobs D.R., Silventoinen K. (2007). Comparison of body mass index, waist circumference and waist/hip ratio in predicting incident diabetes: A meta-analysis. Epidemiol. Rev..

[2] Eckel R.H., Kahn S.E., Ferrannini E., Goldfine A.B., Nathan D.M., Schwartz M.W., Smith R.J., Smith S.R. (2011). Obesity and type 2 diabetes: What can be unified and what needs to be individualized?. Diabetes Care.

[3] Stice E., Spoor S., Bohon C., Veldhuizen M.G., Small D.M. (2008). Relation of reward from food intake and anticipated food intake to obesity: A functional magnetic resonance imaging study. J. Abnorm. Psychol..

[4] Martens M.J., Born J.M., Lemmens S.G., Karhunen L., Heinecke A., Goebel R., Adam T.C., Westerterp-Plantenga M.S. (2013). Increased sensitivity to food cues in the fasted state and decreased inhibitory control in the satiated state in the overweight. Am. J. Clin. Nutr..

[5] Chechlacz M., Rotshtein P., Klamer S., Porubska K., Higgs S., Booth D., Fritsche A., Preissl H., Abele H., Birbaumer N. (2009). Diabetes dietary management alters responses to food pictures in brain regions associated with motivation and emotion: A functional magnetic resonance imaging study. Diabetologia.

[6] Carnell S., Gibson C., Benson L., Ochner C.N., Geliebter A. (2012). Neuroimaging and obesity: Current knowledge and future directions. Obes. Rev..

[7] Vidarsdottir S., Smeets P.A., Eichelsheim D.L., van Osch M.J., Viergever M.A., Romijn J.A., van der Grond J., Pijl H. (2007). Glucose ingestion fails to inhibit hypothalamic neuronal activity in patients with type 2 diabetes. Diabetes.

[8] Kullmann S., Heni M., Veit R., Scheffler K., Machann J., Haring H.U., Fritsche A., Preissl H. (2015). Selective insulin resistance in homeostatic and cognitive control brain areas in overweight and obese adults. Diabetes Care.

[9] Adam T.C., Tsao S., Page K.A., Hu H., Hasson R.E., Goran M.I. (2015). Insulin sensitivity and brain reward activation in overweight Hispanic girls: A pilot study. Pediatr. Obes..

[10] Jastreboff A.M., Sinha R., Lacadie C., Small D.M., Sherwin R.S., Potenza M.N. (2013). Neural correlates of stress- and food cue-induced food craving in obesity: Association with insulin levels. Diabetes Care.

[11] Drummen M., Dorenbos E., Vreugdenhil A.C., Raben A., Westerterp-Plantenga M.S., Adam T.C. (2018). Insulin resistance, weight and behavioural variables as determinants of brain reactivity to food cues—A preview study. Am. J. Clin. Nutr..

[12] Figlewicz D.P., Evans S.B., Murphy J., Hoen M., Baskin D.G. (2003). Expression of receptors for insulin and leptin in the ventral tegmental area/substantia nigra (VTA/SN) of the rat. Brain Res..

[13] Behary P., Miras A.D. (2014). Brain responses to food and weight loss. Exp. Physiol..

[14] Frank S., Heinze J.M., Fritsche A., Linder K., von Feilitzsch M., Konigsrainer A., Häring H.-U., Veit R., Preissl H. (2016). Neuronal Food Reward Activity in Patients with type 2 diabetes with improved glycaemic control after Bariatric Surgery. Diabetes Care.

[15] Journel M., Chaumontet C., Darcel N., Fromentin G., Tome D. (2012). Brain responses to high-protein diets. Adv. Nutr..

[16] Min D.K., Tuor U.I., Koopmans H.S., Chelikani P.K. (2011). Changes in differential functional magnetic resonance signals in the rodent brain elicited by mixed-nutrient or protein-enriched meals. Gastroenterology.

[17] Li J., An R., Zhang Y., Li X., Wang S. (2012). Correlations of macronutrient-induced functional magnetic resonance imaging signal changes in human brain and gut hormone responses. Am. J. Clin. Nutr..

[18] Leidy H.J., Ortinau L.C., Douglas S.M., Hoertel H.A. (2013). Beneficial effects of a higher-protein breakfast on the appetitive, hormonal and neural signals controlling energy intake regulation in overweight/obese, “breakfast-skipping,” late-adolescent girls. Am. J. Clin. Nutr..

[19] Griffioen-Roose S., Smeets P.A., van den Heuvel E., Boesveldt S., Finlayson G., de Graaf C. (2014). Human protein status modulates brain reward responses to food cues. Am. J. Clin. Nutr..

[20] Fogelholm M., Larsen T.M., Westerterp-Plantenga M., Macdonald I., Martinez J.A., Boyadjieva N., Poppitt S., Schlicht W., Stratton G., Sundvall J. (2017). PREVIEW: Prevention of Diabetes through Lifestyle Intervention and Population Studies in Europe and around the World. Design, Methods and Baseline Participant Description of an Adult Cohort Enrolled into a Three-Year Randomised Clinical Trial. Nutrients.

[21] Lang P.J. (1995). The emotion probe. Studies of motivation and attention. Am. Psychol..

[22] Plasqui G., Soenen S., Westerterp-Plantenga M.S., Westerterp K.R. (2011). Measurement of longitudinal changes in body composition during weight loss and maintenance in overweight and obese subjects using air-displacement plethysmography in comparison with the deuterium dilution technique. Int. J. Obes..

[23] Matthews D.R., Hosker J.P., Rudenski A.S., Naylor B.A., Treacher D.F., Turner R.C. (1985). Homeostasis model assessment: Insulin resistance and beta-cell function from fasting plasma glucose and insulin concentrations in man. Diabetologia.

[24] Stunkard A.J., Messick S. (1985). The three-factor eating questionnaire to measure dietary restraint, disinhibition and hunger. J. Psychosom. Res..

[25] Institute of Medicine Committee on Military Nutrition Research (1999). The Role of Protein and Amino Acids in Sustaining and Enhancing Performance.

[26] Choi S., Disilvio B., Fernstrom M.H., Fernstrom J.D. (2009). Meal ingestion, amino acids and brain neurotransmitters: Effects of dietary protein source on serotonin and catecholamine synthesis rates. Physiol. Behav..

[27] Growdon J.H., Cohen E.L., Wurtman R.J. (1977). Effects of oral choline administration on serum and CSF choline levels in patients with Huntington’s disease. J. Neurochem..

[28] Kondoh T., Mallick H.N., Torii K. (2009). Activation of the gut-brain axis by dietary glutamate and physiologic significance in energy homeostasis. Am. J. Clin. Nutr..

[29] Uematsu A., Tsurugizawa T., Uneyama H., Torii K. (2010). Brain-gut communication via vagus nerve modulates conditioned flavor preference. Eur. J. Neurosci..

[30] Avena N.M., Rada P., Hoebel B.G. (2009). Sugar and fat bingeing have notable differences in addictive-like behaviour. J. Nutr..

[31] Harris A., Hare T., Rangel A. (2013). Temporally dissociable mechanisms of self-control: Early attentional filtering versus late value modulation. J. Neurosci..

[32] Neseliler S., Hu W., Larcher K., Zacchia M., Dadar M., Scala S.G., Lamarche M., Zeighami Y., Stotland S.C., Larocque M. (2018). Neurocognitive and hormonal correlates of voluntary weight loss in humans. Cell Metab..

[33] Hare T.A., Malmaud J., Rangel A. (2011). Focusing attention on the health aspects of foods changes value signals in vmPFC and improves dietary choice. J. Neurosci..

[34] Deckersbach T., Das S.K., Urban L.E., Salinardi T., Batra P., Rodman A.M., Arulpragasam A.R., Dougherty D.D., Roberts S.B. (2014). Pilot randomized trial demonstrating reversal of obesity-related abnormalities in reward system responsivity to food cues with a behavioural intervention. Nutr. Diabetes.

[35] Ochner C.N., Laferrere B., Afifi L., Atalayer D., Geliebter A., Teixeira J. (2012). Neural responsivity to food cues in fasted and fed states pre and post gastric bypass surgery. Neurosci. Res..

[36] Neill B.V., Bullmore E.T., Miller S., McHugh S., Simons D., Dodds C.M., Koch A., Napolitano A., Nathan P.J. (2012). The relationship between fat mass, eating behaviour and obesity-related psychological traits in overweight and obese individuals. Appetite.

[37] Murdaugh D.L., Cox J.E., Cook E.W., Weller R.E. (2012). fMRI reactivity to high-calorie food pictures predicts short- and long-term outcome in a weight-loss program. NeuroImage.

[38] Luo S., Romero A., Adam T.C., Hu H.H., Monterosso J., Page K.A. (2013). Abdominal fat is associated with a greater brain reward response to high-calorie food cues in Hispanic women. Obesity.

[39] Jastreboff A.M., Lacadie C., Seo D., Kubat J., Van Name M.A., Giannini C., Savoye M., Todd Constable R., Sherwin R.S., Caprio S. (2014). Leptin is associated with exaggerated brain reward and emotion responses to food images in adolescent obesity. Diabetes Care.

[40] Grosshans M., Vollmert C., Vollstadt-Klein S., Tost H., Leber S., Bach P., Bu¨hler M., von der Goltz C., Mutschler J., Loeber S. (2012). Association of leptin with food cue-induced activation in human reward pathways. Arch. Gen. Psychiatry.

[41] Rosenbaum M., Sy M., Pavlovich K., Leibel R.L., Hirsch J. (2008). Leptin reverses weight loss-induced changes in regional neural activity responses to visual food stimuli. J. Clin. Investig..

[42] Cazettes F., Cohen J.I., Yau P.L., Talbot H., Convit A. (2011). Obesity-mediated inflammation may damage the brain circuit that regulates food intake. Brain Res..

[43] Felger J.C., Treadway M.T. (2017). Inflammation Effects on Motivation and Motor Activity: Role of Dopamine. Neuropsychopharmacology.

[44] Heni M., Kullmann S., Preissl H., Fritsche A., Haring H.U. (2015). Impaired insulin action in the human brain: Causes and metabolic consequences. Nat. Rev. Endocrinol..

[45] Tiedemann L.J., Schmid S.M., Hettel J., Giesen K., Francke P., Buchel C., Brassen S. (2017). Central insulin modulates food valuation via mesolimbic pathways. Nat. Commun..

[46] Westerterp-Plantenga M.S., Lejeune M.P., Nijs I., van Ooijen M., Kovacs E.M. (2004). High protein intake sustains weight maintenance after body weight loss in humans. Int. J. Obes. Relat. Metab. Disord..

[47] Westerterp-Plantenga M.S., Kempen K.P., Saris W.H. (1998). Determinants of weight maintenance in women after diet-induced weight reduction. Int. J. Obes. Relat. Metab. Disord..

[48] Rideout C.A., McLean J.A., Barr S.I. (2004). Women with high scores for cognitive dietary restraint choose foods lower in fat and energy. J. Am. Dietetic Assoc..

[49] Moreira P., de Almeida M.D., Sampaio D. (2005). Cognitive restraint is associated with higher intake of vegetables in a sample of university students. Eat. Behav..

