# Supplementary material for: Associations of Brain Reactivity to Food Cues with Weight Loss, Protein Intake and Dietary Restraint during the PREVIEW Intervention
Source: Nutrients. 2018 Nov 15;10(11):1771. doi: 10.3390/nu10111771 (PMC6266251; doi:10.3390/nu10111771)
Supplement: Supplementary file 1 [file nutrients-10-01771-s001.pdf]

Online Supporting Material

Supplemental figure 1. Participant flowchart

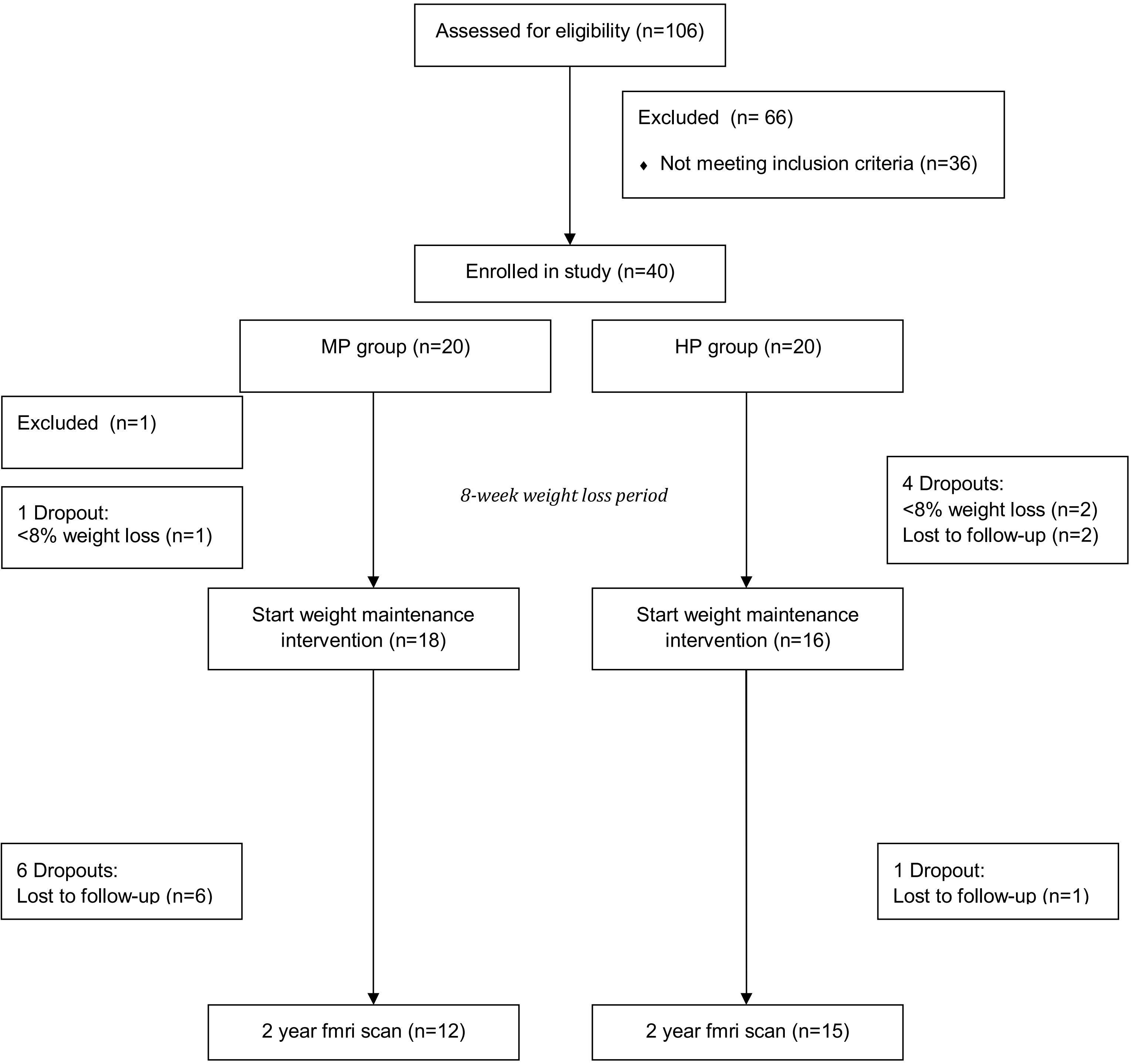

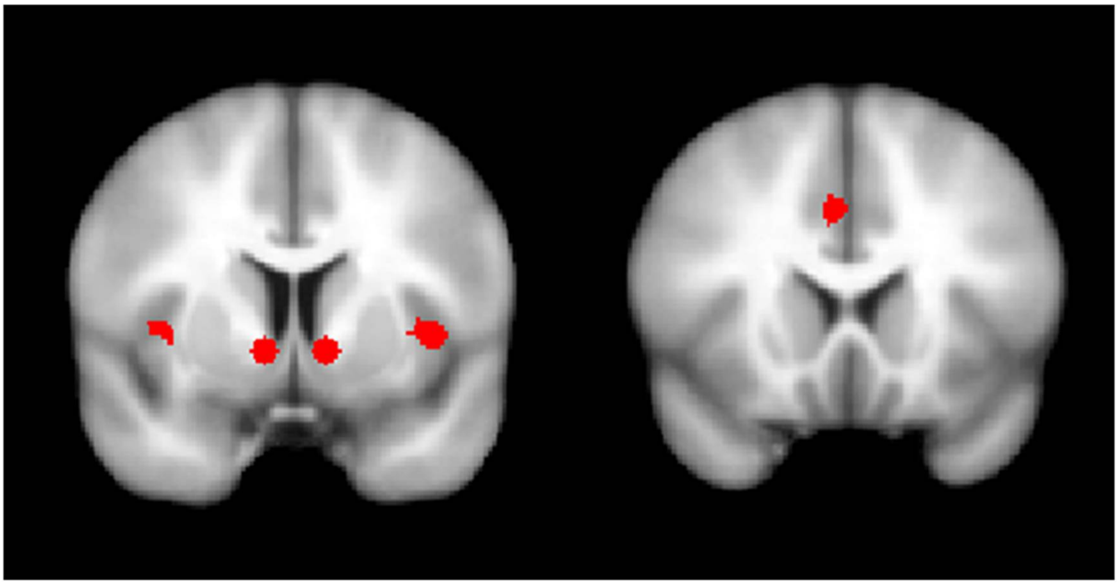

Supplemental figure 2. *A priori* selected regions of interest

Regions were selected from Drummen et al. (2018; AJCN) based on significant associations brain reactivity to food cues and insulin resistance. ROIs included the right and left insular cortex, the left and right nucleus accumbens and the right anterior cingulate.

Online Supporting Material

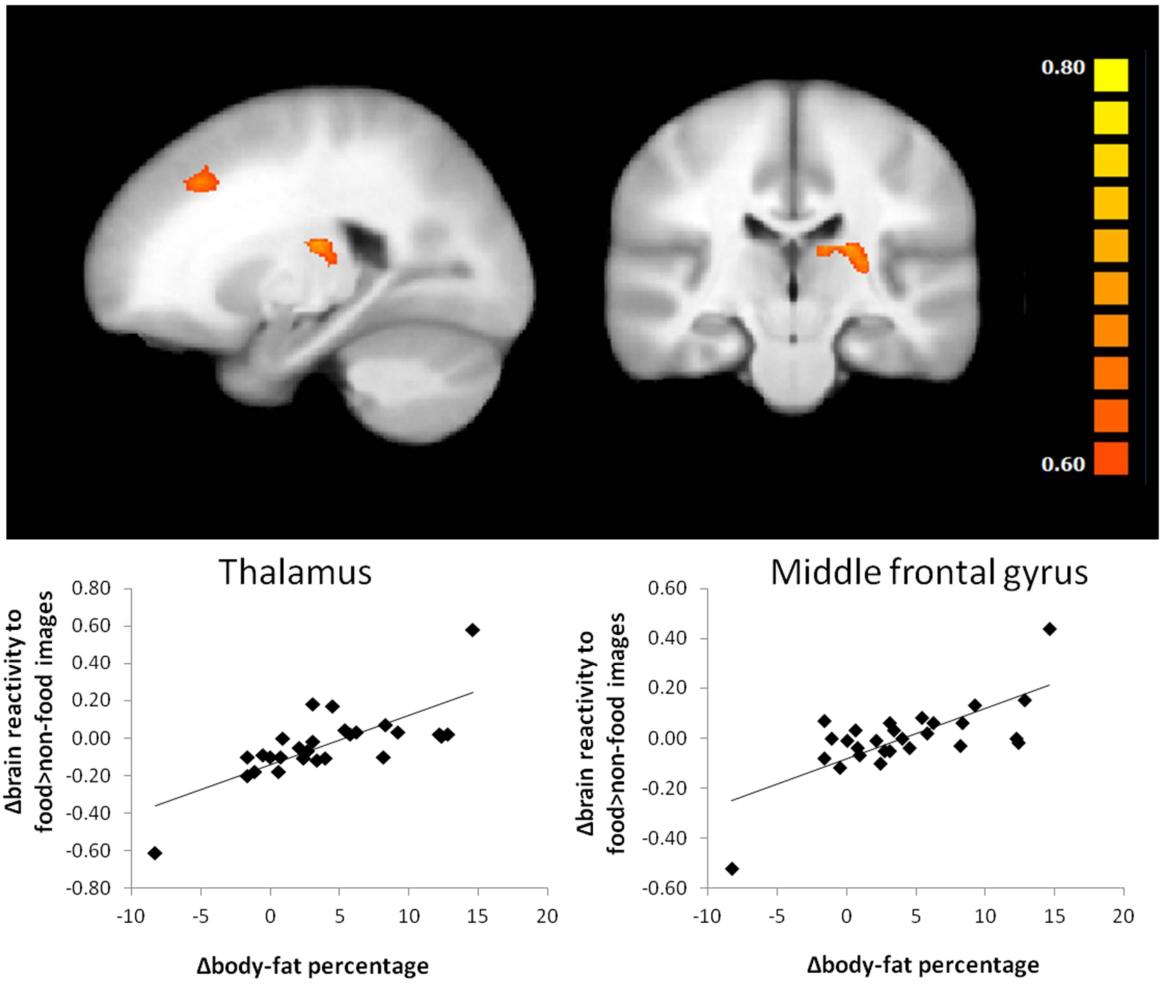

Supplemental figure 3. Whole brain contrast map of brain regions with significant associations between changes in food>non-food brain activation and changes in body-fat percentage. Positive associations are shown in orange (P<0.005, corrected for multiple comparisons). Scatter plots of changes in body-fat percentage and changes in extracted food>non-food BOLD response are shown below.

Online Supporting Material

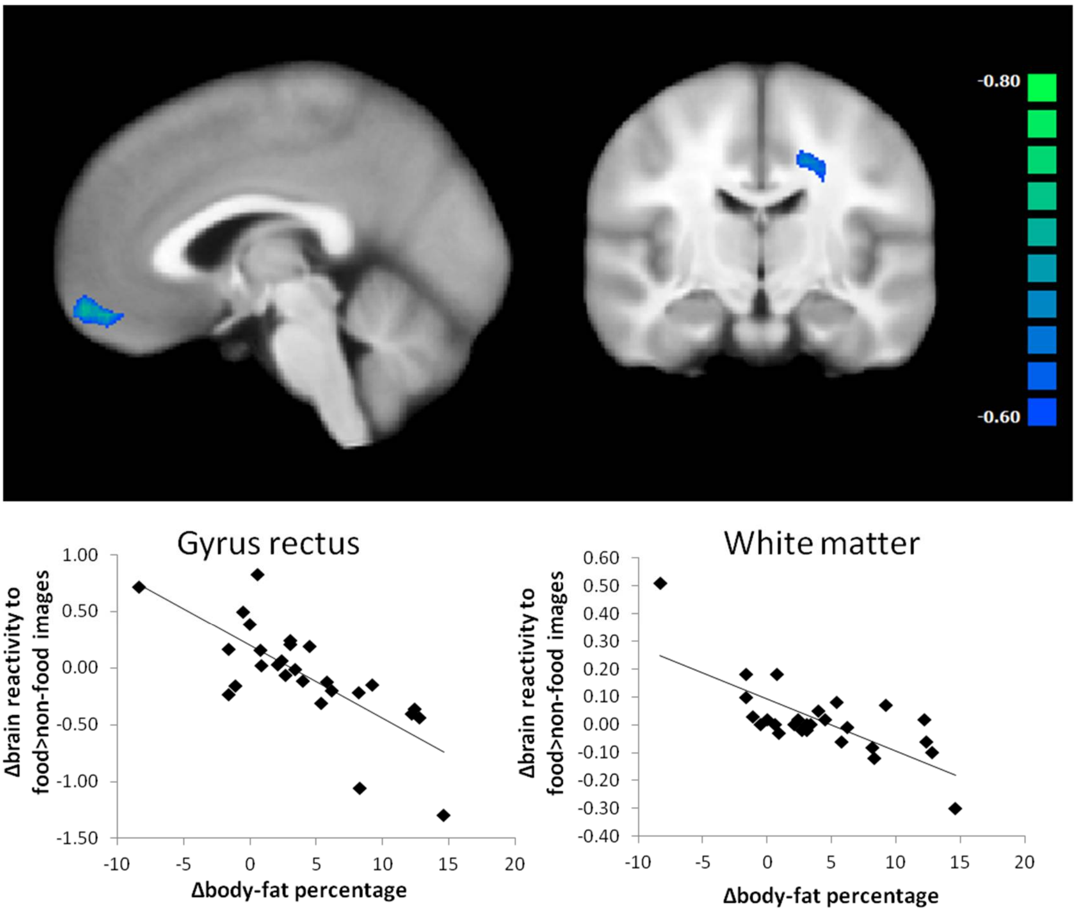

Supplemental figure 4. Whole brain contrast map of brain regions with significant inverse associations between changes in food>non-food brain activation and changes in body-fat percentage. Inverse associations are shown in blue ($P<0.005$, corrected for multiple comparisons). Scatter plots of changes in body-fat percentage and changes in extracted food>non-food BOLD response are shown below.

Online Supporting Material

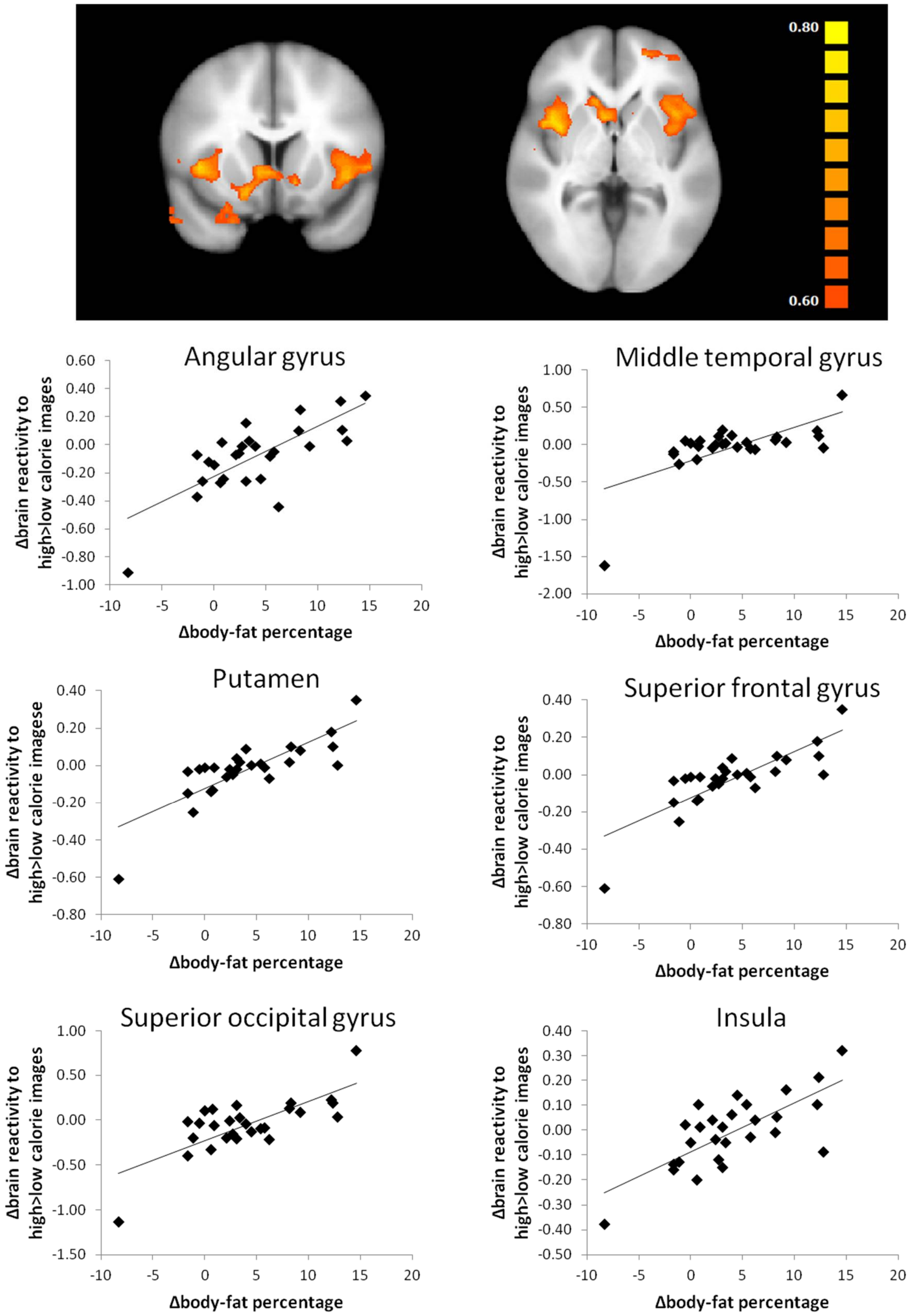

Online Supporting Material

Supplemental figure 5. Whole brain contrast map of brain regions with significant associations between changes in high>low calorie images brain activation and changes in body-fat percentage. Positive associations are shown in orange (P<0.005, corrected for multiple comparisons). Scatter plots of changes in body-fat percentage and changes in extracted high>low calorie images BOLD response are shown below.

Online Supporting Material

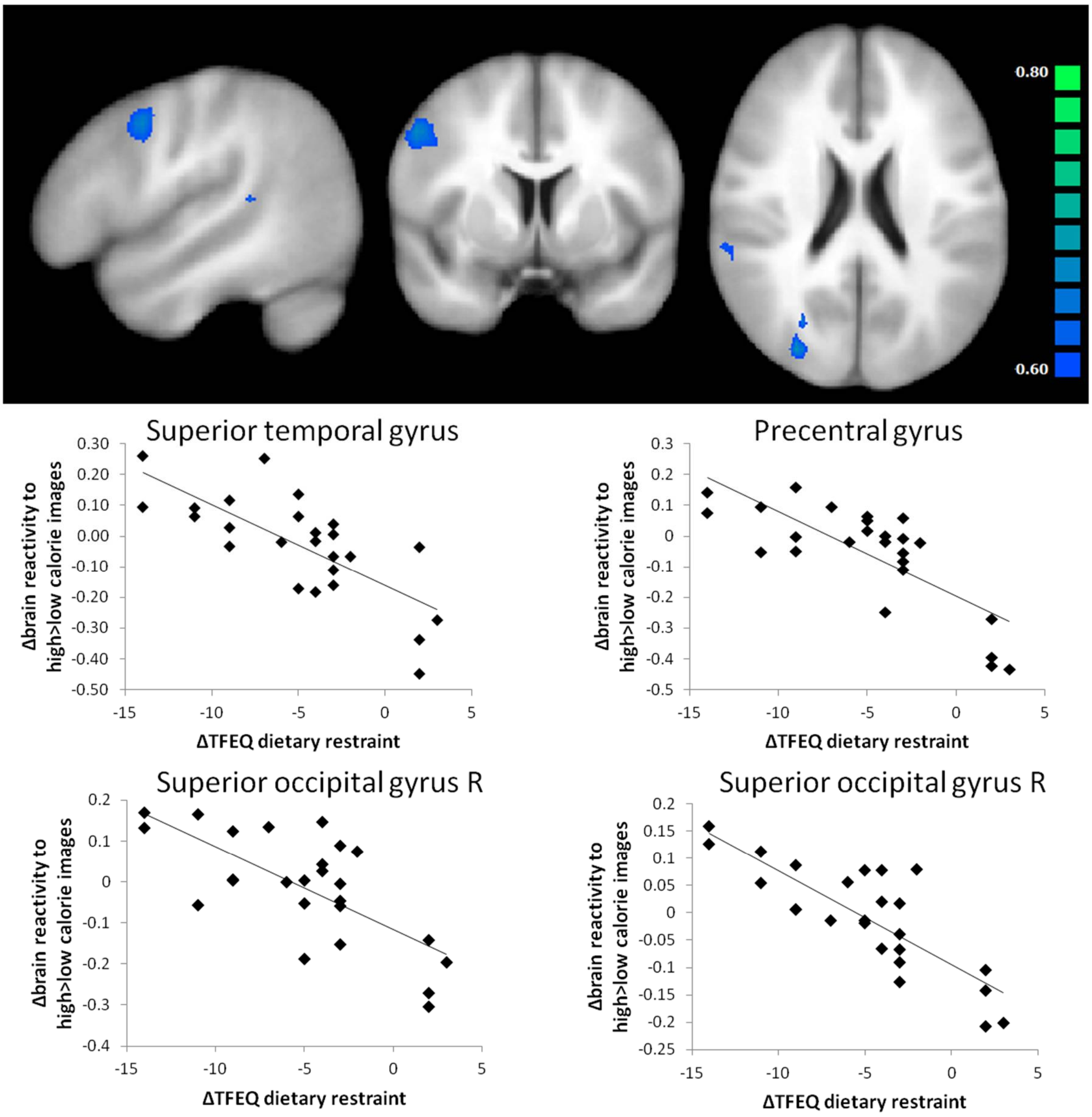

Supplemental figure 6. Whole brain contrast map of brain region with significant inverse associations between changes in high>low calorie images brain activation and changes in TFEQ dietary restraint (factor 1). Inverse associations are shown in blue ($P<0.005$, corrected for multiple comparisons). Scatter plot of changes in TFEQ dietary restraint and changes in extracted high>low calorie images BOLD response are shown below.
